# Mums Alone: Exploring the Role of Isolation and Loneliness in the Narratives of Women Diagnosed with Perinatal Depression

**DOI:** 10.3390/jcm10112271

**Published:** 2021-05-24

**Authors:** Billie Lever Taylor, Louise M. Howard, Katherine Jackson, Sonia Johnson, Nadia Mantovani, Selina Nath, Antoaneta Y. Sokolova, Angela Sweeney

**Affiliations:** 1 Division of Psychiatry, Faculty of Brain Sciences, University College London, Maple House, 149 Tottenham Court Road, London W1T 7NF, UK; s.johnson@ucl.ac.uk; 2 Health Services and Population Research Department, Institute of Psychiatry, Psychology and Neuroscience, Kings College London, De Crespigny Park, London SE5 8AF, UK; louise.howard@kcl.ac.uk (L.M.H.); s.nath@ucl.ac.uk (S.N.); 3 Population Health Sciences Institute, Faculty of Medical Sciences, Newcastle University, Baddiley-Clark Building, Richardson Road, Newcastle NE2 4AX, UK; kat.jackson@newcastle.ac.uk; 4 Population Health Research Institute, St George’s, University of London, Cranmer Terrace, London SW17 0RE, UK; nmantova@sgul.ac.uk; 5 Population, Policy and Practice Research & Teaching Department, UCL Great Ormond Street Institute of Child Health, 30 Guilford Street, London WC1N 1EH, UK; 6 Lived Experience Advisory Group, University College London, Maple House, 149 Tottenham Court Road, London W1T 7NF, UK; mumsaloneadvisorygroup@gmail.com

**Keywords:** perinatal, mental health, loneliness, social isolation, intersectionality, thematic analysis

## Abstract

In this study, we explore the role that isolation and loneliness play in the narratives of women diagnosed with perinatal depression. Isolation and loneliness are increasingly seen as risk factors for depression, including in the perinatal period, but little is known about whether, and in what ways, women themselves associate isolation or loneliness with perinatal distress. Based on the thematic analysis of semi-structured interviews with fourteen mothers in England, we found that women often connected feelings of depression during and after pregnancy to feeling dislocated from their previous identities and relationships. Women felt lost, confined to their homes, and often unsupported by their partners and families. However, fears of being judged to be inadequate mothers made it difficult for women to make authentic connections with others or to express negative feelings, increasing isolation and depression. We drew on the intersectionality theory to illustrate how the intersect between motherhood and other aspects of women’s identities (being young, single, deprived and/or from an ethnic minority) could leave some women particularly isolated and marginalised. Our conclusions emphasise the need to challenge social constructions of the good/bad mother, advocate for social change to lessen pressures on mothers, and develop support that addresses women’s interpersonal contexts and social networks.

## 1. Introduction

The perinatal period, including pregnancy and the first year after childbirth, is increasingly seen as a time when women are at a heightened risk of experiencing psychological distress. The greatest focus in the research literature has been on perinatal depression. The findings from systematic reviews indicate that around one in six women experience depression during pregnancy [1] and around one in five do so in the first three months after giving birth [2]. Perinatal depression has been associated with a range of serious outcomes for women and their infants [3,4].

The high prevalence of distress at this time can seem paradoxical because, across many cultures, having a baby is expected to be a fulfilling event in a woman’s life. Becoming a mother is commonly seen as an ‘achievement’, which confers on women a recognised status and identity in society, at least for those in normative family and social set-ups. However, research, mostly among mothers in the Global North, has shown that for some having a baby not only represents a ‘gain’, but can also result in a complex sense of ‘loss’ of one’s former life [5,6,7,8,9]. Unrealistic normative expectations of motherhood and strong culturally informed views about how women should feel and behave as ‘good mothers’ have also been found to contribute to distress [5,7].

Women’s relationships and social networks undergo changes in the perinatal period, and women can become lonely and isolated. Social isolation is seen as resulting when people have, or perceive themselves to have, limited social contact [10]. Loneliness is subjective, related to an unpleasant feeling of having inadequate social relationships. In early work, Weiss [11] (1973) argued that loneliness consists of the following two key aspects: ‘emotional loneliness’ (absence of a close attachment with another person) and ‘social loneliness’ (lack of integration into a social network). Loneliness and social isolation are generally considered to be multifaceted, overlapping constructs that are also related to other concepts like social support.

A small qualitative study of first-time mothers in the UK found that they had all felt lonely in the transition to motherhood [12]. While the mothers in this study were fairly homogenous (e.g., they were all from professional backgrounds and were all married or cohabiting), they linked their loneliness to making unfavourable self-comparisons with perceived mothering norms, reduced social contact, and a lack of empathy from their partners and other mothers. Other studies have focused on more marginalised groups, highlighting experiences of isolation among teenage, lone and immigrant mothers [13,14,15]. Feelings of loneliness and isolation have also been reported by mothers outside of the Global North [16].

There is growing evidence that isolation and loneliness are major risk factors for depression, including in the perinatal period [17,18]. Such findings suggest a potential benefit from developing perinatal interventions that seek to strengthen women’s support networks to help reduce their distress [19]. In the general population, social isolation and loneliness are increasingly seen as priority areas when supporting people with mental health difficulties [17], and pregnancy and the period after childbirth, when women come into increased contact with healthcare professionals, may be an opportune time to intervene to prevent or alleviate depression.

However, little is known about whether, and in what ways, women themselves consider social isolation or loneliness to be connected to perinatal distress. Qualitative studies with women experiencing postnatal depression have depicted them as “enveloped in unbearable loneliness” [20] (p. 168), “detached and removed from those around them” [6] (p. 182) and, in the case of migrant mothers, experiencing isolation, lack of social support and “a deep sense of loneliness”, sometimes in contrast to what they perceive as a more connected motherhood within their own cultures [21] (p. 8). The intersectionality theory emphasises how factors like gender interact with other social categories, such as class and race, to influence the exclusion and marginalisation of groups or individuals in society [22], and this too seems important to consider in the perinatal context, where isolation, loneliness and distress may not be experienced equally by all women [23]. However, to our knowledge there has been no published research directly exploring the ways in which loneliness and isolation feature in women’s narratives of perinatal depression, or how this may vary for different women.

Our study therefore sets out to explore this, based on qualitative interviews with women from diverse backgrounds who had been treated for perinatal depression. We did not attempt to ‘fit’ women’s stories into a particular model of loneliness or isolation, but instead sought to identify the ways in which women’s narratives demonstrated that loneliness and isolation played a role in their distress. Our aim was to contribute to understanding perinatal distress, using an intersectional lens to consider how this may differ for women in different social groups, and helping inform the development of appropriate support to meet mothers’ needs.

## 2. Materials and Methods

This study was part of a wider research programme called stakeholders’ views and experiences of perinatal mental health care (STACEY) in which qualitative, semi-structured interviews were conducted with 52 women aiming to explore experiences of perinatal mental health services in England [24,25,26]. Ethics approval was granted by the National Health Service (NHS).

### 2.1. Participants

Purposive sampling ensured that women varied in their sociodemographic backgrounds and mental health service use. Inclusion criteria required that women were aged 16 or over, had a 6–9 month old baby at the time of interview and had accessed NHS support for a mental health difficulty during or after their most recent pregnancy. Women who did not speak English were not included due to a lack of resourcing for interpreters. While the wider study included women with a range of diagnoses, we selected for this analysis all participating women who had reported at interview that they had received a diagnosis of perinatal depression (*n* = 14). To retain a focus on perinatal depression we did not include women who reported additional diagnoses. These 14 women were recruited from 5 NHS healthcare providers across London and the south of England.

### 2.2. Data Collection

Women were recruited from NHS services. Eligible women were initially approached by a mental health clinician involved in their support. Clinicians passed contact details of women who were interested in the study to the lead researcher (first author, BLT), who then approached them directly. Potential participants were informed that participation was entirely voluntary and not connected to support in anyway. Written informed consent was obtained.

Semi-structured interview schedules were developed for the wider study by the first author in consultation with the wider study team. These were amended following review by a lived experience advisory group (LEAG) convened for the wider study, consisting of people with experience of perinatal mental health difficulties, and revised again following five pilot interviews. As the wider study focused on experiences of services, interviews included questions about women’s experiences of support for their perinatal mental health. However, women were also asked for their accounts of the distress they had experienced during and after pregnancy. Example questions included the following: Can you start by telling me a bit about your pregnancy? How were things after the birth? Women were not directly asked in interviews about loneliness or isolation, but rather our analysis focused on exploring how these featured spontaneously in their accounts of depression. Women were also asked for basic socio-demographic and diagnostic information.

Interviews, lasting around one hour on average, were conducted between June 2015 and March 2017 in women’s homes, except where women preferred alternative settings (one woman was interviewed in a café and one in an NHS service). The first author carried out 11 of the 14 interviews. Two interviews were conducted by a member of the wider study’s LEAG and one by a Masters student (with the first author also present). The use of a semi-structured interview schedule helped ensure consistency across interviewers. To further aid trustworthiness, each interviewer also wrote up and shared reflexive notes after each interview and debriefed with the first author. All interviews were audio-recorded, transcribed and anonymised.

### 2.3. Data Analysis

We drew on social constructionist theory [27], taking the position that women’s subjective experiences of motherhood are created through social discourse. However, we also acknowledge that these are experienced as real, and that structural and material inequalities are important to women’s experiences too [28]. Interviews were analysed using the principles of thematic analysis, allowing for similarities and differences to be identified across participants [29].

The study was informed by experiential knowledge throughout [30]. Data analysis was jointly led by the last author (AS), who is a survivor researcher with experience of using perinatal mental health services, and the first author, who is a mother and clinical psychologist with experience of working in perinatal mental health services. To enhance rigour, these two authors ran a series of data analysis workshops with three women with relevant lived experience from the wider study’s LEAG, and (separately) with qualitative experts (see Table 1). Our final analytic account consisted of inductive, data-driven analysis of the qualitative interviews interpreted with wider experiential input from the LEAG. The participation of people with lived experience in the data analysis helped us consider women’s narratives from differing standpoints and disrupt traditional boundaries between ‘researcher’ and ‘participant’ [31]. Braun and Clarke’s [29] (2006) six steps of thematic analysis were followed, as follows: (i) The first author read all transcripts. In addition, KJ, NM and AS read three transcripts each, while the three LEAG members (including AYS) read a summary of every transcript that included direct quotations. Observations/reflections on the data were discussed in workshops 1a, 1b and 2a (see Table 1); (ii) the first author re-read the transcripts and systematically arranged segments of text into codes (assisted by NVivo analysis software); (iii) codes were assembled into themes to create a provisional coding frame; (iv) provisional themes were discussed (workshops 2a and 2b) and the coding frame was updated; (v) a comprehensive thematic framework was finalised collaboratively with the LEAG members and qualitative experts and the implications of the findings were discussed (workshops 3a and 3b); (vi) findings were then written up.

## 3. Results

Table 2 shows the characteristics of the 14 women included in this study. Just over half (*n* = 8) were white British, and half (*n* = 7) were married or living with a partner. Five were first-time mothers, five had some degree of children’s social services involvement (support from local authorities who have a duty to safeguard children who may be at risk of harm) and four had received acute mental health support during the perinatal period (admission to an inpatient psychiatric hospital or intensive crisis support at home). All of the names are pseudonyms.

We present our analysis in the following two overarching themes: (1) dislocated self; and (2) dislocated relationships. Each of these include the following separate sub-themes: (1a) what have I become? (1b) stigma, shame and the isolating narrative of the good/bad mother; (2a) difficulties in family relationships and bonds; (2b) nobody to care for me; and (2c) going it alone. These themes are not entirely discrete but instead sometimes overlap, informing and reinforcing one another.

### 3.1. Dislocated Self

#### 3.1.1. What Have I Become?

The narratives of several women in our study connected their feelings of depression to experiencing a dislocated sense of self after having a baby and, in some cases, during pregnancy too. While the women’s distress arguably extended beyond loneliness or isolation, feelings of dislocated identity clearly included a strong sense of loneliness and desolation, as the mothers described themselves becoming confined to their homes with their babies, isolated from the wider world, and disconnected from their past lives and social networks. A dislocated self was particularly evident in the narratives of first-time mothers who had stopped working or taken maternity leave to have a baby.

Lisa, a highly educated married white British mother, who described herself as “an older-ish first-time mum”, said she had a good network of friends locally who also had babies and “loads of support from my parents and my husband”. However, she nevertheless described having “lots of lonely moments” after giving birth, explaining, “I felt isolated, even if I haven’t actually been isolated”. She began to feel low and associated this with the sudden change in her life after giving birth, describing herself as, “Terrified of having a day with no plans”. She struggled to feel connected, integrated and capable at home with her baby, no longer having a routine or role in which she felt competent.

“If you’ve been-, if you have been working, you’ll be going to work every day, you had a role, you’ve been there, you’ve been amongst these people, you’ve been doing that thing. And suddenly, you’re by yourself with this baby, you don’t know what to do with your baby. Really, all day, you just think, ‘Oh, what do I actually do?’ And it, yes, does-, it is quite a lonely thing, actually.”

While some women, like Lisa, had planned pregnancies, others had become pregnant unexpectedly, or had felt pressured into it, and this could make the change to their identity particularly difficult. Lyla, a highly educated Arab British woman living with her husband, described how having children had:

“never been something I’ve particularly wanted or yearned for. I just kind of did it because it was like, you know-…when you come from an Arab or Asian background, you just get harassed for years, ‘When are you having kids? When are you having kids?’”.

Lyla had to stop working to look after her baby and found it became harder to go out socially. She began to long for her previous life, struggling to establish a new sense of self.

“I just kept thinking about all the things that I couldn’t do anymore and wishing for the life I once had...And also, it’s about like, identity. It’s like, sometimes even when she is in bed, it’s like I don’t know what to do. It’s like, apart from eat or watch TV, it’s like, I’m trying to think of things that-, what did I used to do for enjoyment? You know. And I’m trying to remember, what did I used to do?”

A dislocated sense of self was not only experienced by married, highly educated mothers. The narrative of Lottie, a young single white British mother, living in local authority housing, also gave a powerful sense of isolation and loneliness in the context of a loss of her prior life. Lottie described how she was, “so happy before I got pregnant”. However, then Lottie’s partner left her on finding out she was pregnant, and Lottie had to give up her job to look after her baby:

“You lose the man that you were with. You’ve become pregnant, so you end up doing it all on your own. And you haven’t got anyone with you. And you haven’t got a job anymore. So you end up losing your whole life in one big blow”.

Lottie explained that, “Before, I was so confident and outgoing and it was like, ‘Hey, where’s the party?’ And now, it’s just, you just feel lost, in a way.” Lottie felt her body shape had changed too, making her feel like “an obese monster”. While Lottie said her partner “stopped contact…and decided to move on to a new relationship”, she was left struggling to look after their baby alone. Lottie felt that parenthood had little impact on her partner’s life or social status, whereas for her the impact was profound. The combination of being a young, single mother, with no job, and an appearance she no longer liked signified for Lottie a new social status, which left her feeling ashamed and directionless; the intersect of motherhood with age, relationship status and work prospects left her feeling she had shifted into a disadvantaged social position. While Lottie did not explicitly use the words ‘lonely’ or ‘isolated’ in her account, she described struggling to go out, withdrawing from others, and feeling increasingly disconnected.

“It’s raining outside. It’s miserable. There’s nothing to do. And I feel like, you can look at yourself sometimes and just go, what a mess. I don’t want anyone to see me today. I don’t want anyone to come to the door. I’ve had enough…I still have bouts of that, where I look at myself and I cry. I’m just like, what has happened to me? What have I become?”

It was conspicuous that the language used by mothers like Lisa, who had “lots of lonely moments” but also had extensive resources to draw on, including a friendship and family network she found supportive emotionally and practically, gave the impression of loneliness as transient. By contrast, for mothers like Lottie, who had experienced a relationship breakdown and increased material deprivation in motherhood, their accounts suggested they perceived their isolation and loneliness as more permanent and engulfing. In this way, disparities in women’s social and material circumstances intersected with motherhood to affect their experiences.

#### 3.1.2. Stigma, Shame and the Isolating Narrative of the Good/Bad Mother

It was clear from women’s narratives that many experienced a sense of failure or inadequacy that could prevent them from connecting to others. The mothers described feeling under pressure—from themselves, partners, family members, other mothers, and wider societal narratives—to take on the role of a primary caregiver and to be, as Lottie put it, “a perfect mum”. They then experienced feelings of hopelessness when they saw themselves as falling short of this ideal, or when their expectations of motherhood clashed with the reality. Among participating women, this included, for example, not feeling love towards their babies, having outbursts of aggression or anger, not being able to breastfeed, feeling unable to maintain a home or their personal appearance whilst caring for a new baby, or a general a feeling of being unable to cope.

The mothers’ beliefs that they were (or would be seen as) inadequate or different could lead to them avoiding others, including other mothers. This could compound their ‘social loneliness’ [11] or difficulty integrating into social networks. Lottie, for instance, explained how:

“You’ll find that it’s hard to try and see people that are in the same position…When you get offered the chance to see other mums-…quite a lot of them are generally, ‘Look at my baby, I’m so happy. My baby, Antoine, sleeps through the night, he’s perfect.’”

Lottie’s comments seemed also to suggest a class divide, where she felt isolated by the dominant middle class narratives of women in her local groups. Above all, Lottie said she was, “constantly worried that they’re going to judge you”. However, as Lottie also found it hard to maintain her previous friendship group once she was no longer working, she was left isolated, “Suddenly, you’ve got no one, you’re on your own, you hardly see anyone and you become a bit of a hermit”.

When the mothers did manage to make meaningful connections with others who they perceived were struggling in similar ways, however, it could be very reassuring. Lauren, for example, a single black Caribbean British mother, took great comfort from meeting other mothers in a similar position to her on a mother and baby unit (MBU).

“Just knowing that there were other mums, it was just like the biggest comfort ever. I just felt like, oh my gosh, I’m not the only one…because I had friends at the time and they were so in love with their babies and so kind of like, you know, their baby was their world and then I’m feeling the complete opposite and it just made me feel so bad, like I was really crazy. And then just coming to [the MBU] and seeing that, you know, there are other mums that’s just like me.”

Among our participants, the mothers who were single, young, deprived, or from ethnic minority backgrounds (i.e., those who were mothering in what they experienced as more disadvantaged social positions), seemed to experience a particularly acute fear of being judged to be ‘bad mothers’. Yet the narratives of mothers in other circumstances suggested that they too experienced shame connected to anxieties that they were inadequate mothers. There was a sense of loneliness in the mothers’ beliefs that they were alone in their feelings, and ought not admit to them, preventing honest, authentic interactions with others.

For example, Susie, a married white British mother, felt “really disappointed” after finding out she was expecting twin boys and then was “ashamed of that feeling”. She described how she tried to hide how she felt through fear she would be seen as a “bad mother”. Similarly, another married white British mother, Laura, explained how she was “ashamed” for feeling depressed and “not being able to cope”. Although she knew “intellectually that it doesn’t mean I’m a bad mum”, she could not fully accept this and, as a result, felt unable to share how she was feeling, explaining “Very few people know that I, you know, have depression”. This was the case for Mira too, a mixed-race Muslim mother, who kept her struggles “private, you know, because not everyone understands or is willing to hear”.

The accounts of some women, in particular two women, June and Anaya, who both identified as black African and Muslim, suggested that the wider stigma around mental health difficulties in their culture could also increase isolation. June and Anaya each said that, in their cultures, having depression (whether you were a mother or not) was seen as “Your fault, whatever, you’ve done a mistake.” (June). These two mothers appeared already very socially isolated, across the following multiple interlocking axes: they were each seeking asylum in England, neither was living with a partner, both were away from their wider family networks, each lived in insecure housing, and they had little access to resources to provide for their children. The stigma they described within their communities exacerbated the inequalities they experienced and led them to retreat further from those with whom they might otherwise have sought connection.

“You know, depression, what I’m going through…in Africa, the difference is like you are not okay, that person is not okay…That’s why I move away a little bit from people also. You know, it’s ashamed…I don’t want them to know about my situation.” (Anaya)

### 3.2. Dislocated Relationships

#### 3.2.1. Difficulties in Family Relationships and Bonds

The women’s need for nurturing personal relationships was clearly evident across all their accounts, with their experiences of depression, isolation and loneliness strongly rooted in their interpersonal contexts. It was conspicuous how often depression was linked to relationship turmoil, particularly between women and their partners. The two were often closely interwoven in the mothers’ accounts, as illustrated by Nicole, a mother from a black Caribbean British background who had separated from her husband and who described how they:

“was constantly arguing when we was together, so it was hard to differentiate between our kind of bad relationship and the depression.”

The women’s relationships with their partners, and sometimes wider family members too, went through a period of transition with the arrival of a new baby, which could provoke difficulties. Some women described an increased sense of disconnect, or lack of understanding from their partners. One mother described threats of violence from her partner. In several cases, couple relationships had ended in the perinatal period. Nicole felt her husband had struggled to understand her increased vulnerability during and after pregnancy and could not accept the change in their relationship.

“He just couldn’t get his head around it. He didn’t understand what’s the problem. You know, you were such a strong person…It wasn’t like he wasn’t a nice person or like, he wasn’t a good person. He just didn’t get his head around somebody who is strong, who is able to do everything, that everybody depended on, being the other way around. Me dependent on him.”

Fiona, a white British mother, similarly felt her husband “doesn’t really understand mental health”. In other cases, women felt their partners struggled with having to share attention once they had children. For example, Susie explained how her husband “had quite a strong reaction to suddenly being number four in the pecking order from having gone from number one and the centre of my universe.” The relationship difficulties and breakdowns left the women feeling less able to draw on their partners for support or empathy, increasing their ‘emotional loneliness’ in Weiss’s [11] definition, through feeling a lack of close attachment. This could be compounded when women experienced conflict in their wider family networks too. Emma, a single white British mother whose children had been taken into foster care, described her own mother as her main source of support, but also said her mother had been abusive and neglectful to her during and after pregnancy, meaning she could not feel confident in her availability.

Several women also described difficulties bonding with their babies, for example feeling “nothing”, “numb”, “terrified of them” or like their baby “wasn’t a part of me”. For some mothers, a baby’s need to be close to them could feel uncomfortable, even threatening, yet a lack of closeness also resulted in high levels of distress. As Emma explained:

“[My baby] used to have to sleep on my chest and, because I didn’t want him near me, it was really hard having him on my chest. So he used to just lay there and scream in pain and I used to just sit in another part of the room and just cry.”

The emptiness, guilt and shame that the mothers experienced in these cases also contributed to the feelings of disconnect, particularly given the context of the good/bad mother narrative. At the same time, the women’s connections with their babies could also make them feel less alone. Some spoke about their love for their babies ‘keeping them going’. Sometimes closeness with a baby filled an emptiness, depicted in women’s accounts almost as a substitute for a couple relationship. In Emma’s case, for example, she described how when she eventually tried co-sleeping with her baby, “For the first time, I wasn’t in my bed alone, you know”.

#### 3.2.2. Nobody to Care for Me

The difficulties in family relationships, coupled with trouble connecting to other mothers, in part because of the divisive impact of the good/bad mother narrative, contributed to many mothers feeling they lacked adequate support from those around them. The women felt they were expected to take primary responsibility for meeting their baby’s needs, yet—as Nicole’s account highlighted—often felt more “dependent” on others and a need to feel mothered themselves. As Lisa put it, “I just wanted to be looked after.” Lisa described herself as so exhausted that she was “like a little husk”. The mothers’ feelings of exhaustion and overwhelm could make it difficult for them to find the energy to interact with others or socialise. Across the interviews, it was very common for mothers to connect their feelings of depression to the relentlessness of motherhood.

Anaya noted how in Africa, where she was from, mothers usually had considerable support from their wider family networks, in contrast to her experience in England.

“If it’s back home now, you know, your parents, you give them your children sometimes. Two days, three days, they would be with your parents. You have a little rest. But here there’s nobody”.

Nicole similarly spoke about feeling that, “You don’t have anybody”. Half the participants were not living with a partner and, in each case, the mother had taken on the main caregiving responsibility, often with little or no support from anyone else.

However, in some cases women with partners, or with larger family or social networks, could also feel alone and unsupported. This was the case for Lyla, for example, who felt distressed that her husband did not help her care for their baby.

“That’s essentially part of the problem, kind of feeling like I’m on my own, basically…I might as well-, I could just be a single mum and I wouldn’t notice”.

The accounts of mothers who felt they had little help caring for their babies and nobody to care for them contrasted sharply with those mothers who described having supportive networks, which they could draw on to lessen their distress and feelings of being alone. For example, Victoria, a white British mother felt “a little bit like I broke” after she was left alone with her new baby while her husband went away for work. She described “going a little bit stir crazy from just lots and lots of baby contact and not a lot of adult contact”. However, her feelings of distress and isolation were alleviated once her social support increased.

“My husband managed to be sent home a bit early…And also I was kind of lucky in that a few friends were on annual leave and things and they came and helped out with [my baby] just so that I could get a bit more sleep.”

Similarly, Laura—who found it hard be open with her friends about her mental health but otherwise related her depression to physical health complications rather than loneliness or isolation—said that, when she struggled, her husband would “Take over and he’ll let me sort of stay in bed a bit longer”.

Although the women could feel “mind-numbingly bored and isolated…stuck at home all day”—as Carlie, an older white British mother put it—many also longed for rest, respite or alone time. Some women wanted to be by themselves, they wanted peace or an escape. Others wanted alone time with their babies or their immediate family in order to bond. However, what was desired in each case was a nourishing type of aloneness or isolation, in a safe, calm, environment, away from the noise, stresses and overwhelming responsibilities of their lives. Lauren, for example, described how it was her stay on an MBU that finally enabled her to bond with her baby because she felt cared for herself in a safe place that was “kind of like family”. In this context, aloneness or isolation was not a negative, but could feel restorative and healing. As Lauren explained:

“Just shutting the world out and just it being me and her was the main thing. I could just concentrate only on her. Oh, baby. Yes, it being me and her and getting to know each other, just having that time for each other was the main thing, I think.”

Women who had little support from their family or social networks sometimes relied more on professional support to help them feel cared for. Emma, for example, who did not have reliable family support had asked her clinician to be her baby’s godmother. Empathic, supportive relationships with healthcare professionals, who were often described as resembling friends or family members, were seen as an important source of connection. However, these relationships needed to be enduring and empowering, otherwise mothers could feel a sense of abandonment and loss once the support ended. For example, although Lauren really valued the MBU support she received, she described becoming distressed again soon after she was discharged and ultimately having to be readmitted. Similarly, Nicole said she formed a very strong bond with her midwife, who she saw as “in between a mum and a sister”. However, she then felt abandoned when her midwife stopped coming.

“Then my midwife stopped and she was kind of like, like somebody that I really relied on, if you know what I mean…and, I just, I don’t know, it just, it made me feel, I don’t know, like I’d had a loss. You know what I mean? Like I’d lost something…I was already bad. It just, made it a lot worse.”

#### 3.2.3. ‘Going it Alone’

Finally, several women’s narratives depicted them as battling on singlehandedly, despite the challenges they faced. Lottie, for example, described feeling as though she was “in a battle because you’re constantly fighting to stay on top. You’re constantly fighting to try and be happy”. An impression of the mothers ‘going it alone’ seemed to depict them as isolated, but also resilient. It could be seen in the narratives of the women who felt unsupported by their partners or wider networks, but was particularly evident among those mothers who saw themselves as parenting outside of the norms, for example because they were young or single.

Emma described her belief that she was seen as a bad mother in part because she did not match societal norms of what a good mother should be: “You’re a young mum. You have two children close in age. That gives it all away.” While Emma accepted that she had struggled to care for her children, she felt she had been unfairly expected, as a woman, to take naturally to motherhood, “They think we just love it all…the motherly instinct”, but she explained, “I’m a single mum, you know. And it’s not just going to happen by myself…But I was left to do it all by myself”. However, while Emma clearly felt isolated and unsupported, her account also conveyed a sense of strength and resistance. She described herself as proud for struggling on by herself, seeking to prove that, despite everything, she could succeed. Although she did not have custody of her children, she felt that by persevering she had managed to bond with them, particularly her oldest son. She described how:

“We pulled through, me and [my son] together. He’s still in my care-, well he was at the time, you know, still alive and kicking. And I should be proud of myself for that.”

The intersection of motherhood with being young and single in Emma’s account seemed to be connected to feeling marginalised, but also to being strong and distinctive, almost as if she sought to harness her feeling of going it alone to carve out a unique, valued identity and resist the discourses that seemed to be against her. While custody loss was intensely painful, she attempted to turn her sense of dislocated self and aloneness into a positive force, helping her also to find some sense of ‘togetherness’ with her son. Similarly, Lottie’s account of aloneness, although clearly a source of overwhelming distress, seemed also to define her as distinctive and resilient.

“No matter how tired you are, you crack on with it, and no matter how much there is to do, you crack on with it. You can either do it miserable and sad and be depressed about it, or you can put some music on like I’ve been doing and just crack on with it. Do a little dance”.

## 4. Discussion

In this qualitative analysis we explored how loneliness and isolation play a role in the accounts of women diagnosed with perinatal depression. We centralised experiential knowledge in our study, carrying out first-person interviews with women and then analysing their accounts collaboratively with a group of people with relevant lived experience. This ensured that wider experiential knowledge shaped our understanding of the women’s data and that multiple standpoints, experiential and theoretical, informed our interpretation of the findings. This process was grounded in the notion that the interpretation of data varies depending on the standpoint of the analyst [32]. For instance, in their study of service users’ experiences of psychiatric detention, Gillard et al. [33] (2010) found that the analysis and interpretation of mainstream researchers tended to focus on processes and procedures, whilst service user researcher accounts focused more on emotions and experiences. In addition, our analysis highlights how occupying multiple disadvantaged statuses affected the mothers’ sense of loneliness and isolation indicating that the impact of intersecting identities was qualitatively different from the impact of any single identity on loneliness and isolation. The mothers’ identity was intertwined with categories of gender, race/ethnicity, relationship status and class, which they continuously negotiated in everyday social contexts.

### 4.1. Summary of Findings

Based on the interviews with 14 women treated for perinatal depression, we found that mothers often connected their feelings of depression during and after pregnancy to a sense of being dislocated from their previous lives, identities, and relationships. Some explicitly used the words loneliness and isolation to describe their experiences, while others talked about feeling lost, alone, or having nobody. Although the experiences varied, the accounts of all the women informed or related to at least one theme. The women described changes to their identity, associated with a loss of employment, independence, routine, sleep and body shape, as well as changes to their social lives, relationships and friendships. A sense of dislocation arguably extended beyond loneliness and isolation, but the women clearly described themselves as isolated from wider social networks, confined to their homes by the mothering role, and struggling to connect to others or integrate into social networks (‘socially lonely’ [11]) for fear they would be seen as falling short of the ideal ‘good mother’. While struggles with identity, relationships and mothering norms have also been described by perinatal women not diagnosed with depression [12], our participants clearly connected their sense of dislocated self and relationships to their feelings of depression in intricate ways.

The women’s relationships, especially with their partners, underwent transition in the perinatal period, sometimes resulting in conflict, breakdown and even abuse. Many women felt that they were left to care for their babies alone, seen automatically as the primary caregiver, with little support or respite and a lack of close empathic attachment (‘emotionally lonely’; [11]). By contrast, the mothers with supportive networks and partners with whom they shared parental responsibilities, tended to describe loneliness in more transient terms. Yet, an important finding was that women could also feel lonely and isolated despite having people around them if they didn’t feel supported and understood in the way they needed. Although a new baby could make women feel less alone, others felt more alone in the absence of supportive adult company, and some struggled to bond with their infants. Nevertheless, isolation was not only viewed negatively. In some contexts, for example if a woman felt she was in a safe, supportive wider environment, having time to herself, or time alone with her baby, could be restorative and nurturing. Some women also harnessed their aloneness to define themselves as strong, resilient and distinctive.

### 4.2. Setting the Findings in Context

Our study reinforces previous findings that having a baby can result in a complex sense of ‘loss’ for women [5,6,7,8,9]. Feminist scholars in particular have connected postnatal depression to losses and inequalities in women’s lives following childbirth, such as an increasingly unequal division of household work, a gendered employment market, loss of occupational status and identity, increased confinement to the home, and a high burden of care in which women are also held responsible for their infants’ developmental outcomes, e.g., [34,35]. In our study, loss was clearly apparent in the women’s accounts, particularly among first-time mothers who had given up their jobs or taken time away from work to have a baby. More than forty years on, our themes of ‘dislocated self’ and ‘dislocated relationships’ resonate with Oakley’s [36] (1980) seminal sociological study of childbirth in which she argued that a change in self, particularly the loss of paid work roles and of previous more equal or joint relationships with partners, can be akin to a form of bereavement which can lead to significant mental distress.

The themes of ‘dislocated self’ and ‘dislocated relationships’ also have resemblances with Mauthner’s [7] (1999) relational approach to postnatal depression in which she argues that depression results when mothers feel unable to experience, communicate and validate their needs and feelings within supportive relationships and an accepting wider social and cultural context. She sees relational issues, and women’s sense of agency (or lack of it) within these relational settings as key to understanding postnatal depression. Mauthner [7] also views depression as linked to a disconnect between the expectations of motherhood—how mothers think they should be in order to be ‘good mothers’—and the reality in which women often feel inadequate and unsupported. However, as in our study, Mauthner notes that isolation is also two-way, with women who feel depressed actively withdrawing from relationships and networks and ‘silencing the self’ [37] through fear of being a burden or being judged as ‘bad mothers’. This self-reinforcing relationship between mothering norms, isolation and depression is also noted by Knudson-Martin and Silverstein [38] (2009) in a qualitative data synthesis of postnatal depression. They found, across studies from a range of different countries, that social constructions of the ‘good mother’ made it difficult for women to express negative emotions and to have these validated by others, provoking feelings of incompetence that precipitated isolation from others.

In our analysis, the binary narrative of the ‘good/bad mother’ featured strongly across the women’s accounts. It organised how women thought about, interpreted, and performed their mothering roles. This ‘master discourse’ [39] was, to some extent, reinforced by the women themselves, who used it to define themselves as worthy or unworthy of moral respect. Yet it was experienced as monolithic and coercing, creating rigid expectations around women’s feelings, behaviours and supposedly innate mothering abilities, and provoking a sense of lonely distress and disconnect from others when women felt unable to meet these expectations. As found by Knudson-Martin and Silverstein [38], expressing negative feelings was seen as incongruent with social constructions of motherhood. This acted to silence and isolate women, who at times became overwhelmed with feelings of incompetence, making authentic connection with others difficult.

Some women did, however, make meaningful connections with other mothers who they felt were in similar situations to themselves, such as in the case of one mother who connected with others on a psychiatric mother and baby unit. When this happened, it was experienced as comforting and motivating. Similarly, Mauthner [40] (1995) found in her research that forming honest friendships with other mothers was key to reducing women’s feelings of depression, enabling them to challenge and resist cultural norms of motherhood through shared experiences. Mauthner [40] suggests that peer relationships may be even more important than couple relationships at this time. However, in our research, difficulties in couple relationships were often presented by women as central to their distress.

We found that women could also open up to and gain important support from relationships with professionals. As in previous research, when women’s own families did not provide adequate support, they appeared more likely to view other people as family [41]. However, as professional relationships were often not enduring, their temporality could ultimately increase women’s feelings of loneliness or abandonment, particularly among those who relied more on these relationships because they were already isolated or marginalised. This reinforces previous research that has shown how people accessing mental health support may experience a painful sense of loss and insecurity if a therapeutic relationship ends [42].

A strength of our study is that the heterogeneity of our participants allowed us to draw on the intersectionality theory to consider how women’s positions within different social categories could influence their feelings of depression, isolation and loneliness. The intersectionality theory is principally concerned with how people’s locations across multiple interdependent social categories (e.g., gender, ethnicity, age, and class) can affect experiences and result in privilege or marginalisation [43]. In our study, although there are themes that were common to all participants, our findings show how ethnicity, relationship status, age, and the material and subjective components of class and deprivation could intersect with motherhood to affect women’s experiences. Motherhood could be a location for increased disadvantage or privilege, depending on different elements of intersectional identity, leaving some women more vulnerable than others to sustained loneliness, isolation and marginalisation.

In particular, while fears of being judged to be a ‘bad mother’ were apparent across most of the women’s accounts, our findings point to a perception among women that certain characteristics (e.g., being young, single, or deprived) are more likely to be associated with deviant, deficient motherhood, increasing the feelings of rejection and isolation. For example, one woman who was parenting alone, believed that being young with two children close in age led others to reject her as an inadequate mother. Another woman described how she lost her partner and job and gained weight during pregnancy, shifting her from an identity with which she had felt happy into a new disadvantaged social status, as a young, single, unemployed mother. The intersect of age, relationship status and work prospects with motherhood left her feeling an overwhelming sense of failure, constraint, and aloneness. Other women found themselves situated in disadvantaged social positions as mothers because of factors such as their status as asylum seekers, which left them cut-off from wider family networks, and struggling to access housing or other resources they felt they needed to provide for their children. The stigma around mental health, particularly within minority ethnic groups, could further exacerbate women’s marginalisation.

In this way, our work also has similarities with Abrams and Curran’s [23] (2011) study of identity among low-income mothers experiencing postnatal depression, in which they found that postpartum depression and poverty could converge to create a ‘troubled identity’ of maternal failure. Nonetheless, Abrams and Curran also found that women sought to resist this identity by portraying themselves as loving, self-sacrificing, and engaged mothers. In our analysis, we also noted how mothers who felt they were parenting outside of the accepted norms in some ways appeared to embrace aloneness and marginalisation, harnessing it to define themselves as strong, resilient and distinctive. This could potentially be viewed as an example of “narrative repair” [44], where those who feel they are prevented from occupying valued social roles because their identities categorise them as inferior, seek to resist or repair this by reidentifying themselves as competent members of the community.

While our study offers insights into women’s experiences, it also had limitations. As outlined, the women were not explicitly asked about loneliness and isolation in interviews, but rather we explored how these concepts featured spontaneously in their accounts, either because mothers directly mentioned them or where they were implicit in their narratives. Further insights could be gained by asking women directly about loneliness and isolation in the context of perinatal depression. While the 14 women we interviewed came from diverse social and cultural backgrounds, they all lived in the south of England, many in London, and some ethnicities were not represented (e.g., Asian). Future research would benefit from interviewing women from a wider range of geographical areas, including rural areas, as well as interviewing women from more ethnicities and those who do not speak English. Finally, future research should seek to explore with women what support they believe might help to reduce their feelings of loneliness or isolation, or strengthen their social networks.

### 4.3. Conclusions and Implications

Our analysis suggests that loneliness and isolation are related to perinatal depression in complex, intricate, and often mutually reinforcing ways. Our findings have important implications. While it is important to be aware that in rare cases mothers can pose a serious threat to their children, our analysis supports the work of those who argue that if we wish to improve the experience of motherhood for women, we must seek to broaden the societal frameworks within which mothers are expected to operate, and challenge the artificial, binary good/bad mother divide [37]. Our research also suggests there is a need to advocate for broader social change to lessen pressures on women and their families, reduce deprivation among mothers, and seek to improve gender equality.

The women’s accounts in our study point to the often social nature of perinatal depression. An implication of this is the importance of developing support for women, which is not only focussed on the individual but also addresses interpersonal relationships, social networks, and wider social structures. While healthcare professionals like midwives increasingly ask women about feelings of depression during and after pregnancy, it may prove helpful also to ask about women’s families and wider social networks and to explore whether women may benefit from support to strengthen these. Our findings support the idea that finding ways to facilitate connections between mothers in the perinatal period, for example through peer support, may be valuable [45,46]. However, this must be done in a way that is sensitive to ethnicity, class, age and relationship status, and recognises that mothers may be fearful of connecting with others. A focus on developing and evaluating interventions focused on couple relationships and wider social networks is likely to have value. The interventions should help parents to identify and expand their ideals of parenthood, including emphasising how cultural norms can affect parents’ feelings of competence. Finally, while our findings suggest that women’s relationships with professionals can also lessen their feelings of isolation and help them feel supported and validated, there is a need to ensure services offer women support in a way that is sustainable and that recognises the human nature of therapeutic interactions.

## Figures and Tables

**Table 1:** Purpose and format of workshops.

Workshop	Purpose	Content
Workshop 1a: three women from the wider study LEAG (including AYS)	To refine the analysis plan.	Guided discussions of: (a) the study; (b) the qualitative analysis plans; and (c) members’ own experiences of the topic.
Workshop 1b: qualitative experts (KJ and NM)	As above	Guided discussions of: (a) the qualitative analysis plans; and (b) three full transcripts circulated prior to workshop.
Workshop 2a: LEAG	To reflect on the women’s narratives, aid theme development and contextualise the emerging analytic account	Guided discussions of: (a) a document, circulated prior to workshop, containing a summary of each woman’s story (fully anonymised) with demographic information and example direct quotations from each interview; and (b) a summary of early and emerging themes, presented during the workshop after part a. Example questions reflected on: How do you understand these mothers’ experiences of isolation, loneliness and depression after reading their stories? What connects their stories? How do they differ? How did these women’s stories connect to, and differ from, your own experiences? How do things like deprivation, age, race and being a single mother or not, affect women’s experiences?
Workshop 2b: qualitative experts	As above for 2a	As above
Workshop 3a: LEAG	To review the final themes and consider study implications.	Guided discussion of: (a) the final thematic account and its interpretation; (b) study implications.
Workshop 3b: qualitative experts	As above for 3a	As above

**Table 2:** Characteristics of participating women (*N* = 14).

Mother’s Name	Age	Living with Partner	Ethnicity ^a^	Social Services Involvement ^b^	Level of NHS Mental Health Support	Highest Qualification	Number of Children
Lottie	<25	No	White British	No	Primary care	Secondary or pre-university	1
Lauren	<25	No	Black Caribbean	Yes	Acute care	Secondary or pre-university	1
Carlie	>40	Yes	White British	No	Primary care	Secondary or pre-university	3 or more
Lyla	30-39	Yes	Arab	No	Primary care	University degree or higher	1
Victoria	30-39	Yes	White British	No	Acute care	University degree or higher	1
Nicole	30-39	No	Black Caribbean	Yes	Secondary care	University degree or higher	2
Fiona	30-39	Yes	White British	No	Secondary care	University degree or higher	2
Anaya	30-39	No	Black African	No	Acute care	Secondary or pre-university	3 or more
Susie	30-39	Yes	White British	Yes	Primary care	Secondary or pre-university	3 or more
Emma	<25	No	White British	Yes	Secondary care	Secondary or pre-university	2
Lisa	30-39	Yes	White British	No	Acute care	University degree or higher	1
June	25-29	No	Black African	No	Secondary care	No qualifications	3 or more
Laura	30-39	Yes	White British	No	Secondary care	University degree or higher	2
Mira	25-29	No	Mixed race	Yes	Secondary care	No qualifications	3 or more

^a^ Input to safeguard children who may be at risk of harm. ^b^ Acute (inpatient or crisis) care, secondary (specialist community) care, or primary (less intensive) care.

## Data Availability

The datasets generated and/or analysed during the current study are not publicly available due to them containing information that could compromise research participant privacy/consent but are available from the corresponding author on reasonable request.
